# NLR and Intestinal Dysbiosis-Associated Inflammatory Illness: Drivers or Dampers?

**DOI:** 10.3389/fimmu.2020.01810

**Published:** 2020-08-11

**Authors:** Jefferson Elias-Oliveira, Jefferson Antônio Leite, Ítalo Sousa Pereira, Jhefferson Barbosa Guimarães, Gabriel Martins da Costa Manso, João Santana Silva, Rita Cássia Tostes, Daniela Carlos

**Affiliations:** ^1^Department of Biochemistry and Immunology, Ribeirão Preto Medical School, University of São Paulo, Ribeirão Preto, Brazil; ^2^Department of Pharmacology, Ribeirão Preto Medical School, University of São Paulo, Ribeirão Preto, Brazil

**Keywords:** NLRs, microbiota, gut dysbiosis, diabetes, inflammatory diseases

## Abstract

The intestinal microbiome maintains a close relationship with the host immunity. This connection fosters a health state by direct and indirect mechanisms. Direct influences occur mainly through the production of short-chain fatty acids (SCFAs), gastrointestinal hormones and precursors of bioactive molecules. Indirect mechanisms comprise the crosstalk between bacterial products and the host's innate immune system. Conversely, intestinal dysbiosis is a condition found in a large number of chronic intestinal inflammatory diseases, such as ulcerative colitis and Crohn's disease, as well as in diseases associated with low-grade inflammation, such as obesity, type 1 and 2 diabetes mellitus and cardiovascular diseases. NOD-Like receptors (NLRs) are cytoplasmic receptors expressed by adaptive and innate immune cells that form a multiprotein complex, termed the inflammasome, responsible for the release of mature interleukin (IL)-1β and IL-18. NLRs are also involved in the recognition of bacterial components and production of antimicrobial molecules that shape the gut microbiota and maintain the intestinal homeostasis. Recent novel findings show that NLRs may act as positive or negative regulators of inflammation by modulating NF-κB activation. This mini-review presents current and updated evidence on the interplay between NLRs and gut microbiota and their dual role, contributing to progression or conferring protection, in diabetes and other inflammatory diseases.

## Introduction

The healthy human intestine is colonized by several microorganisms, including fungi, viruses, and bacteria belonging to different families (1). Studies on the gut microbiome show a high number of bacteria from the Bacteroidaceae, Prevotellaceae, Rikenellaceae, and Ruminococcaceae families in the colon (2). On the other hand, the small intestine is mainly colonized by bacteria from the Lactobacillaceae and Enterobacteriaceae families (3). In recent years, sequencing analysis of the 16S rRNA gene revealed an association between the gut microbiota and inflammatory diseases (4). Changes in the composition of the intestinal microbiota, a process called dysbiosis, play a key role in the pathogenesis of inflammatory diseases, such as rheumatoid arthritis (5), atherosclerosis (6), ulcerative colitis, Crohn's disease (7), and diabetes mellitus type 1 and 2 (8, 9). Accordingly, modulation of the gut microbiota by prebiotics and probiotics, as preventive or therapeutic strategies to mitigate the pathogenesis of inflammatory diseases, has been increasingly investigated (10).

Innate immunity receptors, also called pattern recognition receptors (PRRs), are expressed by several cells and are involved in the recognition of microbial products or endogenous self-molecules. PRRs are key components in the pathogenesis of inflammatory and autoimmune diseases (11, 12). Toll-Like Receptors (TLRs) and NOD-Like Receptors (NLRs) are among the main families that comprise the PRRs superfamily (13). In the process of dysbiosis, the increased pathobiontic bacteria modulates the expression and activation of TLRs, leading to a pro-inflammatory response in the intestine and in extra-intestinal sites (14, 15). On the other hand, NLRs have either beneficial or harmful effects that rely on the antimicrobial factors and pro-inflammatory cytokine profile following gut microbiota activation. This mini review highlights the divergent roles of NLRs in metabolic and inflammatory diseases associated with gut dysbiosis.

## Gut Dysbiosis in Inflammatory Diseases

The intestinal microbiota, when in homeostasis, is directly related to the host's health. The intestinal microbiota influences host metabolism (16), immune system (17, 18), gut microbicide mechanisms (19), and maintains the intestinal barrier (20). Many studies show that environmental factors, such as the use of antibiotics (21, 22), diet (23) and stress (24) can alter the intestinal microbiota, increasing pathobiontic bacteria at the expense of commensal bacteria, a process known as dysbiosis (25). Gut dysbiosis contributes to the development of several autoimmune, inflammatory and metabolic diseases, such as rheumatoid arthritis (RA), inflammatory bowel diseases (IBD), and diabetes mellitus (26, 27). However, in many cases, as in IBD for example, it is not yet known whether dysbiosis is the cause or consequence of the disease (28, 29). The exact role of the gut microbiota in the pathogenesis of RA is not fully understood either. However, germ-free (GF) mice exhibit a delay in the development of RA when compared to the control group (30). In the early stages of RA, a decrease in some commensal bacteria, such as those belonging to the *Bifidobacteria* and *Bacteroides* genus, and an increase in *Escherichia coli* and *Proteus mirabilis* have been reported (31, 32). In addition, RA patients have an increase in *Prevotella copri* as well as in anti-*P. copri* IgA and IgG, suggesting that this bacteria may contribute to the pathogenesis of RA (33).

Inflammatory bowel diseases, such as Crohn's disease (CD) and ulcerative colitis (UC), affect ~3 million people in Europe and the USA, with a high and accelerated incidence in developing countries (34, 35). Although the etiology is still unclear, genetic predisposition and environmental factors, such as diet and use of antibiotics, are triggers of these diseases, characterized mainly by chronic intestinal inflammation (34, 36). In addition, disruption of the epithelial barrier and gut dysbiosis are widely reported in patients and in experimental models of gastrointestinal infections (37, 38) including patients with IBD (39, 40). 16S rRNA metagenomic analysis showed that the microbiota present in the feces of mice with UC is very different from microbiota in the feces of healthy mice, mainly by an increase in species of the phylum Verrucomicrobia and a decrease in Tenericutes in mice with colitis, which correlates with a higher disease score (41, 42). An increase in Enterobacteria is observed in fecal samples from patients with CD (43). *Escherichia* and *Shigella* abundance is also increased in this condition, when compared to healthy individuals. In addition, a reduction of the *Roseburia, Coprococcus*, and *Ruminococcus* genera, which are important butyrate producers, has been reported (44, 45). Analysis of colon biopsies from patients with IBD also shows a decrease in Firmicutes and an increase in Bacteroidetes (46) and patients with IBD exhibit increased biofilm production of strains of *Enterococcus* when compared to strains from the control group (47).

An imbalanced gut microbiota and changes in the intestinal barrier function are also closely linked to the pathogenesis of diabetes mellitus (DM) (48). DM comprises a group of metabolic diseases characterized mainly by chronic hyperglycemia, resulting from impaired secretion and/or insulin functionality (49). In type 1 diabetes (T1D), also called autoimmune diabetes, autoantibodies are present and autoreactive lymphocytes mediate pancreatic β-cell destruction, leading to complete insulin deficiency (50). The impact of the microbiota on the development of T1D was demonstrated using Myd88-deficient non-obese diabetic (NOD) mice bred in pathogen-free (SPF) or germ-free (GF) conditions. Whereas, SPF NOD.Myd88^−/−^ mice are protected from T1D, mice under GF conditions develop T1D, showing that Myd88 protective effects depend on the presence of gut microbiota (51). In this context, many studies have shown differences in the composition of the microbiota between diabetic and non-diabetic patients, suggesting that these changes are associated with the development and severity of T1D (52, 53). Bacterial proteome studies show high enrichment with *Clostridium* and *Bacteroides* proteins in children with T1D, whereas the control group exhibit greater enrichment with *Bifidobacterium* proteins (54). Furthermore, the decrease in lactate- and butyrate-producing species, such *B. adolescentis*, is associated with T1D autoimmunity (55).

In models of type 2 diabetes (T2D), gut dysbiosis aggravates the inflammatory process, increases intestinal permeability and also alters the metabolism of short-chain fatty acids, which are important in insulin resistance (56), in addition to accelerating the development of obesity, retinopathy and nephropathy (57). In patients with T2D, excessive intake of carbohydrates and proteins is associated with an imbalance in the gut microbiota, with an increase in the *Clostridium* genus and a decrease in *Bifidobacterium* spp. and *Lactobacillus*, in addition to glucose intolerance (58). Moreover, in experimental models of T2D, the administration of bacteria of the *Bifidobacterium* genus improves glucose tolerance and confers a protective role in the development of T2D (59, 60). Similarly, the administration of *Bacteroides acidifaciens* decreases insulin resistance and even prevents obesity (61).

Innate immunity receptors, such as NLRs, have a decisive role in protecting the intestinal barrier against various microorganisms from the environment. These receptors also modulate microbial intestinal composition, being associated with the development of inflammatory diseases (62).

## Protective Role of NLRs in Gut Microbiota Homeostasis and IBD

The innate immune system components are the first barrier against infections and recognize cell death, generating a rapid immune response due to the recognition of Pathogen-Associated Molecular Pattern (PAMPs) and Damage-associated molecular patterns (DAMPs), respectively (63). NLRs are part of a variety of innate immunity receptors, located in the intracellular environment, and initiate inflammatory processes. NOD1 and NOD2, central members of NLRs, mainly recognize bacterial peptidoglycan and, thus, induce gene transcription of NF-kB and mitogen-activated protein kinases (MAPKs), activating the expression of pro-inflammatory factors by different cells (13).

The NOD1 receptor detects gamma D-glutamyl-meso-diaminopimelic acid (γ iE-DAP), a peptide found mainly in Gram-negative bacteria, but also in groups of Gram-positive bacteria such as *Listeria* spp. and *Bacillus* spp (64–66). In the absence of NOD1, there is expansion of some intestinal bacteria, such as Clostridiales*, Bacteroides* spp., segmented filamentous bacteria (SFB), and Enterobacteriaceae. The NOD2 receptor detects the muramyl dipeptide (MDP) present in the bacterial peptideoglycan and is the most important receptor in intestinal homeostatic control (67). This receptor controls commensal microbiota and the elimination of pathogenic bacteria in intestinal crypts, minimizing the risk of intestinal inflammation and colorectal cancer (68–70). Interestingly, NOD2 expression depends on the presence of intestinal commensal bacteria, indicating a positive feedback relationship. NOD2 deficiency breaks this homeostatic interaction, resulting in gut dysbiosis, and increased IBD susceptibility (69).

Other NLRs also play an important role in intestinal homeostasis. The activation of NOD-like receptor family-pyrin domain containing 6 (NLRP6), through oligomerization and assembly of proteins–inflammasome complex–activates caspase-1 and leads to the synthesis of IL-1β and IL-18 in the intestinal epithelium (71). The deficiency of NLRP6 in mouse colonic epithelial cells decreases IL-18 levels, promotes gut dysbiosis and increases the risk of colitis (72, 73). IL-18 secreted by epithelial cells stimulates the barrier function and the regeneration of epithelial cells (73). In addition, commensal microbiota itself activates the NLRP6 inflammasome, leading to the production of mucus by goblet cells and antimicrobial peptides, maintaining a healthy composition of the intestinal microbiota (74).

NLRP3, another type of NLRs, is highly expressed in the monocytic lineage (75), and favors a greater production of IL-1β over IL-18, leading to changes in the composition of the intestinal microbiota (76). Under normal conditions, NLRP3 deficient mice exhibit gut dysbiosis associated with an excessive growth of Prevotellaceae and Bacteroidetes (77), whereas the ratio between Firmicutes and Bacterioidetes decreases (78). Unlike other NLRs, NLRP12 has anti-inflammatory effects, inhibiting canonical and non-canonical NF-κB; decreasing the production of inflammatory cytokines, chemokines and tumorigenic factors (79–82), and controlling infection by Gram-negative bacteria (83). NLRP12 deficiency, in a dextran sodium sulfate (DSS)-induced colitis model, promotes colon inflammation, decreases gut microbiota diversity and increases colitogenic bacteria, such Erysipelotrichaceae family, depicting a protective role of NLRP12 in IBD (84).

## Divergent Roles of NLRs and AIM2 in T1D Development

**Figure 1:**
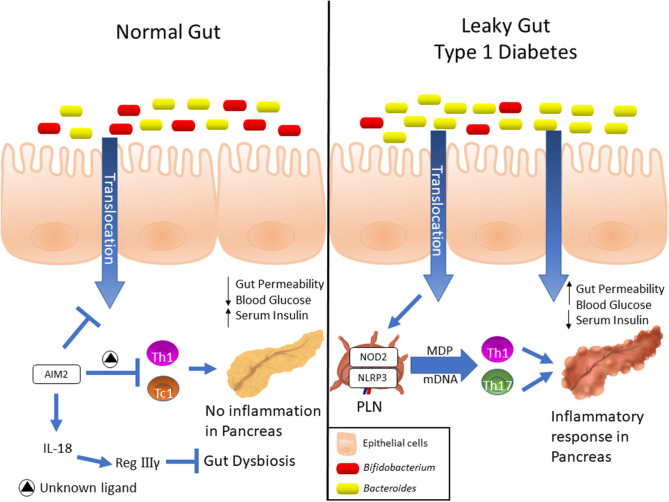
Expression and differential functions of NLRs in Type 1 diabetes development. Elevated AIM2 expression was detected into intestinal mucosa of pre-diabetic mice, and its activation induces the IL-18 release, which in turn, promotes the RegIIIγ production. This mechanism attenuates the gut dysbiosis, reinforces the gut barrier and dampens the Th1 and Tc1 lymphocyte response against insulin-producing β cells, which ultimately protects against T1D. On the other hand, NOD2 recognizes translocated muramyl dipeptide (MDP) from dysbiotic microbiota, and contributes to the activation of Th1 and Th17 lymphocytes in T1D. Finally, upregulation of NLRP3 expression in PLNs was observed in diabetic mice, which is activated in macrophages by recognition of mitochondrial DNA (mDNA), leads to IL-1β production and drives the pathogenic Th17 and Th1 lymphocyte generation, resulting in T1D onset.

In the past few years, several lines of evidence have demonstrated that members of the NLRs family participate in T1D pathogenesis. Recently, we reported that mice lacking NOD2, but not NOD1, are resistant to streptozotocin (STZ)-induced T1D and are unable to induce a Th1 and Th17 immune response in the pancreatic lymph nodes (PLNs) and pancreas. Interestingly, diabetic mice exhibit changes in the composition of the gut microbiota, and this is associated with gut microbiota translocation to PLNs (Figure 1). When these mice are submitted to a broad-spectrum antibiotic treatment, previously to the STZ injections, they do not develop signs of T1D, such as hyperglycemia. Additionally, the administration of the NOD2 ligand, MDP, promotes STZ-induced T1D in antibiotic-treated, STZ-injected wild-type (WT) mice. Our results demonstrate that gut microbiota recognition by NOD2 in the PLNs triggers a proinflammatory response, which induces a Th1 and Th17 cell pathogenic immune response, thus contributing to STZ-induced T1D pathogenesis (Table 1) (85).

**Table 1:** Summary of experimental studies about the role of NLRs in T1D.

**NLR**	**Main findings**	**References**
NOD2	Gut microbiota translocation to PLNs triggers proinflammatory response mediated by NOD2 activation, which contributes to STZ-induced T1D onset; *Nod2*-deficient NOD mice are protected from diabetes development and the protection is most likely mediated by altered gut microbiota	(85, 86)
NLRP3	NLRP3 inflammasome activation by mitochondrial DNA promotes IL-1β release, contributing to the generation of pathogenic Th17/Th1 cells in the PLNs, and increasing T1D susceptibility in STZ-induced T1D model; NLRP3 deficiency in NOD mice inhibits the expression of chemokines and chemokine receptors involved in immune cell migration to pancreatic islets of NOD mice, which protects NOD mice against T1D development.	(87–89)
AIM2	AIM2 plays a protective role in STZ-induced T1D by regulating gut dysbiosis, intestinal permeability, and reducing bacterial translocation to PLNs, which limits the generation of diabetogenic pathogenic Th1 and Tc1 cells.	(90)

STZ-injected WT mice display an increase in different bacteria groups in the gut microbiota, such as Bacteroidaceae family and the *Bacteroides* genus that have been associated with increased susceptibility to T1D in humans (91, 92). These results recapitulate what has been found in type 1 diabetic patients, with the Bacteroidetes phylum, the Bacteroidaceae family, and the *Bacteroides* genus being more commonly found in autoantibody-positive children than in autoantibody-negative peers (55). Other important observation found among type 1 diabetic patients is decreased microbiota diversity, associated with reduced relative abundance of *Bifidobacterium, Roseburia, Faecalibacterium*, and *Lachnospira* (91). These data indicate that gut dysbiosis observed in type 1 diabetic patients may act as an environmental trigger in the development of the disease and that strategies aiming blockade of NOD2 signaling emerge as potential therapies for T1D. Similar results were reported in spontaneous T1D mice model. Non-cohoused NOD.NOD2^−/−^ mice exhibit reduced T1D incidence and a decrease in CD4^+^ IFN-γ^+^/CD8^+^ IFN-γ^+^ (Th1/Tc1) and CD4^+^ IL-17^+^/CD8^+^ IL-17^+^ (Th17/Tc17) T cells in PLNs, indicating that NOD2 activation regulates T1D development by altering the composition of gut microbiota and by modulating the adaptive immune response (86).

Other studies also revealed that NLRP3 is required for T1D pathogenesis. NLRP3 deficiency in NOD mice protects against T1D by inhibiting the expression of chemokines and chemokine receptors involved in immune cell migration to pancreatic islets. NLRP3 deficiency in NOD mice reduces the expression of CCR5 and CXRC3 on T cells and also the gene expression of CCL5 and CXCL10 in pancreatic tissue and these processes occur in an IRF1-dependent manner (87). Additionally, our research group demonstrated that NLRP3 inflammasome activation by mitochondrial DNA (mDNA) promotes IL-1β release by macrophages, contributing to the generation of pathogenic Th17/Th1 cells in the PLNs and to T1D susceptibility in STZ-induced T1D model (Figure 1, Table 1) (88). In accordance, an association study in a north-eastern Brazilian population identified two single-nucleotide polymorphisms (SNPs) in NLRP3, rs10754558, and rs358294199, that are associated with T1D in humans, suggesting that variations in NLRP3 may be a predisposing genetic factor for the development of autoimmune T1D (89).

Another innate immune receptor that results in inflammasome assembly upon its activation, is the DNA sensor absent in melanoma 2 (AIM2) (93, 94). The activation of AIM2 is involved in autoimmune and inflammatory diseases (95). In the STZ T1D model, AIM2 is highly expressed in the ileum at early stages of the disease. Interestingly, AIM2^−/−^ STZ-injected mice display increased T1D incidence, augmented intestinal permeability and bacterial translocation to PLNs, which leads to a proinflammatory response mediated by Th1 and Tc1 cells. When the gut microbiota is depleted by a broad-spectrum antibiotic cocktail before STZ-injections, the increased susceptibility to T1D observed in AIM2^−/−^ mice is abrogated (Table 1). The effects induced by AIM2 activation *in vivo* are mediated by IL-18 release, which favors regenerating islet-derived III gamma (RegIIIγ) production, thus mitigating gut microbiota alterations and reinforcing the intestinal barrier function. Together, our data show that AIM2 activation limits gut microbiota dysbiosis, intestinal permeability and translocation to PLNs, decreasing a proinflammatory response, and conferring protection against T1D (90).

## NLRs Role in Obesity, T2D and Comorbidities

Gut dysbiosis can lead to increased permeability of the intestinal barrier, resulting in low-grade systemic inflammation and metabolic disorders such as obesity, T2D and ischemic stroke (96, 97). Receptors of the innate immunity play a role in systemic inflammation caused by obesity. Mice fed a high-fat diet (HFD) exhibit an increase in colonic inflammation and endotoxemia due to elevated intestinal permeability of the colon mucosa (98). Additionally, increased TLR4 signaling in the colon and activation of NF-κB are observed (98). However, female mice lacking TLR4 display higher risk of developing obesity, but also greater protection to insulin resistance, perhaps due to the lack of TLR4 signaling in important organs for metabolic homeostasis (99). In addition, other studies showed that gut dysbiosis promotes a state of metabolic endotoxemia during obesity, resulting in blood LPS accumulation, metainflammation and insulin resistance through CD14/TLR4 pathway (100–102).

T2D is a chronic metabolic inflammatory condition and is the most common type of diabetes in adults worldwide (103). This disease is initiated by the worsening of pancreatic dysfunction, established when insulin production by β-pancreatic cells cannot keep up with the increase in peripheral insulin resistance (104, 105). Low-grade systemic inflammation accompanies diabetes, with high serum levels of C-reactive protein (CRP), tumor necrosis factor (TNF-α), monocyte chemo-attracting protein-1 (MCP-1) and IL-1β (106, 107). In addition, obesity, aging and other conditions that promote low-grade chronic inflammation are linked to increased risk of developing T2D (108–110). Systemically, the high serum concentrations of IL-6, IL-1β, and TNF-α increase insulin resistance and cause endothelial dysfunction, priming the vascular system to the development of diabetes-related diseases, including systemic arterial hypertension (111). Meanwhile, increased pancreatic IL-1β, IL-6, and IL-8 decrease insulin gene expression in β-pancreatic cells, contributing to increased insulin resistance (112).

A fine balance between the activation of innate NOD1 and NOD2 receptors is crucial for maintaining peripheral insulin resistance. Direct activation of NOD1 receptors through intraperitoneal administration of NOD1 ligand in WT mice leads to an increase in peripheral insulin resistance in up to 6 h (113). In this same study, the activation of NOD1 induced small increases in circulating proinflammatory cytokines. In addition, higher concentrations of inflammatory mediators are observed in cultures of 3T3-L1 fibroblasts differentiated into adipocytes and exposed for 18 h to NOD1 ligands (113). Inflammation of peripheral tissues, especially adipose tissue (114), is a hallmark of T2D and directly contributes to its pathogenesis through adipose tissue dysfunction and subsequent complications in energy homeostasis and intermediate metabolism (115). In this context, double knockout NOD1/NOD2 mice are protected against peripheral insulin resistance and peripheral inflammation observed in the obesity (HFD)-induced T2D model (113).

NOD2 is an innate immunity receptor that recognizes peptidoglycan in the cell wall of bacteria and, therefore, constitutes an important link between gut microbiota and immunity (116). Therefore, NOD2 activation profile may be important in metabolic diseases with immune branches and, therefore, may represent the link in the cross-talk between the gut microbiota and these diseases. The deficiency of NOD2 in mice allows greater translocation of bacteria from the intestine (116). In a model of HFD, mice deficient in NOD2 exhibit greater peripheral resistance to insulin, inflammation of visceral adipose tissue, and higher content of bacterial DNA in the liver (117). HFD increases the Firmicutes to Bacteriodetes ratio and NOD2^−/−^ mice submitted to HFD exhibit dysbiosis, represented by an increase in the number of *Helicobacter* bacteria and in the Peptococcaceae family and reduction of the *Clostridium* genus, when compared to HFD-fed WT mice (117). Fecal transplantation from obese mice to lean GF mice increases total body mass and adipose-tissue mass. Alternatively, fecal transplantation from lean mice to obese GF mice reduces the adipose-tissue mass (118). In this last experimental setup, there is an increase in the number of Bacteriodetes phylum in the gut, which has been related to the production of microbiota metabolites with host modulatory properties, in particular: SCFAs (118). Interestingly, adequate consumption of dietary fiber favors the secretion of SCFAs by the intestinal microbiota. In addition, the activation of the GPR43 receptor in mice, by SCFAs, in M2 macrophages of adipose tissue leads to increased metabolic activity and favors maintenance and homeostasis of healthy adipose tissue, improving metabolic health (119).

## Concluding Remarks

The different categories of NLRs modulate gut dysbiosis-driven extra-intestinal and intestinal inflammatory diseases. The effects of NLRs are diverse and may be either protective or deleterious depending on the immunological context. In the intestine, NLRs regulate gut microbiota composition and translocation by influencing mucus secretion and antimicrobial peptide production, thus playing a key role in the protection against inflammatory bowel diseases, such as ulcerative colitis and Crohn's disease. Alternatively, NLRs also are activated by microbial PAMPs (gut microbiota) or endogenous DAMPs (components from dead or dying cells), which act as negative or positive regulators of the innate and adaptive immunity response and contribute to the susceptibility or resistance to metabolic diseases such as obesity, type 1, type 2 diabetes, and their comorbidities. Thus, the pharmacological modulation of these receptors may represent new therapeutic strategies for these inflammatory and metabolic diseases.

## Author Contributions

JE-O, JL, ÍP, JG, and GM equally contributed to the manuscript writing. JS and RT provided scientific assistance and revised it critically. DC coordinated and reviewed the manuscript. All authors approved the submission and publication.

## Conflict of Interest

The authors declare that the research was conducted in the absence of any commercial or financial relationships that could be construed as a potential conflict of interest.
